# BNIPL is a promising biomarker of laryngeal cancer: novel insights from bioinformatics analysis and experimental validation

**DOI:** 10.1186/s12920-024-01811-z

**Published:** 2024-02-01

**Authors:** Rui Wang, Ying Gao, Shuxin Wen, Xiudong Guo

**Affiliations:** 1 Department of Otolaryngology Head and Neck Surgery, Shanxi Bethune Hospital, Shanxi Academy of Medical Sciences, Tongji Shanxi Hospital, Third Hospital of Shanxi Medical University, No. 99, Longcheng Street, Taiyuan City, Shanxi Province 030032 China; 2 Tongji Hospital, Tongji Medical College, Huazhong University of Science and Technology, Wuhan City, Hubei Province 430030 China; 3 Department of Head Neck and Breast Oncology, Shanxi Bethune Hospital, Shanxi Academy of Medical Sciences, Tongji Shanxi Hospital, Third Hospital of Shanxi Medical University, Taiyuan City, Shanxi Province 030032 China

**Keywords:** Laryngeal cancer, DEGs, BNIPL, Hub gene

## Abstract

**Background:**

Laryngeal cancer (LC) is a malignant tumor with high incidence and mortality. We aim to explore key genes as novel biomarkers to find potential target of LC in clinic diagnosis and treatment.

**Methods:**

We retrieved GSE143224 and GSE84957 datasets from the Gene Expression Omnibus database to screen the differentially expressed genes (DEGs). Hub genes were identified from protein-protein interaction networks and further determined using receiver operating characteristic curves and principal component analysis. The expression of hub gene was verified by quantitative real time polymerase chain reaction. The transfection efficiency of BCL2 interacting protein like (BNIPL) was measured by western blot. Proliferation, migration, and invasion abilities were detected by Cell Counting Kit-8, wound-healing, and transwell assays, respectively.

**Results:**

Total 96 overlapping DEGs were screened out from GSE143224 and GSE84957 datasets. Six hub genes (BNIPL, KRT4, IGFBP3, MMP10, MMP3, and TGFBI) were identified from PPI network. BNIPL was selected as the target gene. The receiver operating characteristic curves of BNIPL suggested that the false positive rate was 18.5% and the true positive rate was 81.5%, showing high predictive values for LC. The expression level of BNIPL was downregulated in TU212 and TU686 cells. Additionally, overexpression of BNIPL suppressed the proliferation, migration, and invasion of TU212 and TU686 cells.

**Conclusion:**

BNIPL is a novel gene signature involved in LC progression, which exerts an inhibitory effect on LC development. These findings provide a novel insight into the pathogenesis of LC.

**Supplementary Information:**

The online version contains supplementary material available at 10.1186/s12920-024-01811-z.

## Introduction

Laryngeal cancer (LC) is a kind of malignancy of head and neck with high incidence, with approximately 1.7 million cases of LC and nearly 90,000 deaths worldwide each year [1]. More than 40% of LC patients with LC are diagnosed at an advanced stage, often with spread to the supraglottic and para glottic space [2, 3]. The 5-year overall survival rate for LC shows continued decline in the last few decades [4]. The occurrence of LC is multifactorial and is related to common carcinogens (tobacco and alcohol) [5]. Radiotherapy, chemotherapy, and surgical resection are the main treatments for LC, but the recurrence rate of advanced LC is 25-50%, and there are still numerous patients with poor prognosis [6, 7]. Therefore, screening the promising biomarkers and therapeutic targets for the early diagnosis of LC has become a hot topic.

Currently, the potential for genomic data sharing plays a crucial role in cancer research [8]. The identification of genes involved in cancer progression holds great promise for medical diagnosis [9]. After retrieving quantitative and clinical gene expression data of LC from the Cancer Genome Atlas and Gene Expression Omnibus (GEO) database, the role of B7-H3 in the diagnosis and prognosis of LC is determined [10]. Based on the robust rank aggregation analysis for the consolidation of gene expression datasets from the GEO database for patients with LC, CDK1, PC24, HOXB7, and SELENBP1 are screened as potential biomarkers of LC [11]. Therefore, searching for potential gene markers as the therapeutic targets for LC is utmost essential.

BCL2 interacting protein like (BNIPL) is a pro-apoptotic gene that can maintain homeostasis between cell proliferation and apoptosis in vivo [12]. There are 2 spliceosomes of BNIPL, BNIPL-1 and BNIPL-2. Overexpression of BNIPL-2 can significantly inhibit tumor growth by regulating molecules related to cell proliferation and apoptosis of hepatocellular carcinoma cells [13]. Additionally, BNIPL-2 can interact with Bcl-2 and Cdc42GAP to regulate cell apoptosis in hepatocellular carcinoma [14]. However, whether BNIPL is involved in the progression of LC remains unknown and need to be further explored.

In this study, we screened out common differentially expressed genes (DEGs) from the GSE143224 and GSE84957 datasets. The hub genes of LC were determined after constructing protein-protein interaction (PPI) network. Then we investigated the role of BNIPL in proliferation, migration, and invasion of LC in vitro. It is hoped that this study will provide novel insights of LC pathogenesis and BNIPL can be a potential target of LC in clinic diagnosis and treatment.

## Materials and methods

### Microarray dataset selection

The gene expression datasets of LC-related genes were downloaded from GEO of National Center for Biotechnology Information (https://www.ncbi.nih.gov/geo/). We searched “Laryngeal Squamous Cell Carcinoma” in the GEO database, and the LC-related microarray datasets, GSE143224 (11 control samples vs. 14 tumor samples) and GSE84957 (9 control samples *vs.* 9 tumors samples) were selected for analysis of this study.

### Identification of DEGs

GSE143224 and GSE84957 datasets were analyzed using Gene Expression Profiling Interactive Analysis (GEO2R; www.ncbi.nlm.nih.gov/geo/geo2r).|logFC| ≥ 2 and adj. *p* ≤ 0.05 were set as the cutoff criteria for selecting DEGs. The identified DEGs were visualized using heat maps and volcano maps, and the samples were normalized and corrected by boxplot.

### Enrichment analysis of DEGs

DAVID (The Database for Annotation, Visualization and Integrated Discovery) (http://david.abcc.ncifcrf.gov/) database was applied to perform Gene Ontology (GO) and Kyoto Encyclopedia of Genes and Genomes (KEGG) pathway enrichment analysis. GO analysis results were divided into molecular function (MF), biological processes (BP), and cellular component (CC). The results with the minimum p value were regarded as the most significantly enriched targets, which were displayed using the enrichment analysis bar chart and enrichment analysis bubble chart.

### PPI network construction and hub genes identification

PPI network were constructed based on STRING online database (https://www.string-db.org/) to explore the interaction network between proteins encoded by DEGs. The confidence interaction score was set at 0.4 as the significance criterion. Visualization of the protein interaction network was carried out using Cytoscape software. The significant modules were screened out from PPI networks using Molecular Complex Detection online tool. The plugin CytoHubba (Version 0.1) of Cytoscape was used to calculate the degree of protein node, and the hub genes were selected according to the connection degree.

### Analysis of hub genes

GO chord plots of hub genes were draw to reveal the relationship between proteins and the changes of the functional pathways. The expression of hub genes in GSE84957 dataset was used as a variable to perform principal component analysis (PCA). Then the expression levels of hub genes in GSE84957 dataset were visualized using ridgeline plots. Above analyis and visualization were performed using R package ggplot2.

The receiver operating characteristic (ROC) curves were generated by Gene Expression Profile Interaction Analysis database (GEPIA; http://gepia.cancer-pku.cn/) to assess the diagnostic accuracy of hub genes. The Box-Plot in Expression-DIY in Expression Analysis from GEPIA2 (http://gepia2.cancer-pku.cn/#index) was conducted to verify the expression of hub genes in LC. After that, Kaplan-Meier plotter (http://kmplot.com/analysis/) was applied to reveal the association between the expression levels of the key gene and the survival of patients with LC. The relationship between the expression level of hub genes and the infiltration of immune cell in LC microenvironment was analyzed using Tumor Immune Estimation Resource database (https://cistrome.shinyapps.io/timer/).

### Cell treatment and transfection

TU212 (iCell-h220), TU686 (iCell-h216) cells and normal laryngeal epithelial cells (NLEC, HUM-iCell-m020) were purchased from iCell Bioscience Inc (Shanghai, China). TU212 and TU686 cell lines were grown in Roswell Park Memorial Institute Medium 1640 (Gbico, New York, USA) supplemented with 10% fetal bovine serum (Gbico) and a 1% penicillin and streptomycin combination (Beyotime, Shanghai, China) at 37 °C with 5% CO_2_. The pcDNA3.1 encoding the BNIPL was used to overexpress BNIPL and constructed by Ribobio (Guangzhou, China). The expression level of BNIPL after transfection in cells was determined by western blot.

### Quantitative real time polymerase chain reaction (qRT-PCR)

Total RNA was extracted by Invitrogen™ TRIzol™ Reagent (Invitrogen, Carlsbad, CA, USA), and reverse transcribed to cDNA using PCR Amplifier. qRT-PCR was performed using ABI7500 quantitative PCR (Applied Biosystems, Foster City, CA, USA) with the following procedures: 95 °C for 10 min for initial denaturation, 95 °C for 10 s for denaturation, 60 °C for 30 s for annealing, and 36 cycles in total. GAPDH was used as an internal reference gene. The Ct values were analyzed by the 2^−ΔΔCt^ method. The experiments were repeated 3 times. Primer (Takara, Otsu, Shiga, Japan) sequence was shown in Table S1.

### Western blot

Protein was extracted from cells using lysis buffer (Beyotime), and the protein concentration was determined by bicinchoninic acid quantitative kit (Thermo Fisher Scientific, Waltham, MA, USA). The protein samples were denatured at 95 °C for 5 min. The samples were then separated on the sodium dodecyl sulfate-polyacrylamide gel electrophoresis and were transferred to the polyvinylidene fluoride membranes. After being blocked with 5% skim milk, the proteins were then incubated with anti-BNIPL (1:2000; Abcam, Cambridge, UK) and anti-GAPDH antibody (1:1000, Abcam) at 4 °C overnight. Subsequently, the membranes were incubated with horseradish peroxidase-labeled goat anti-rabbit IgG (1:5000; Abcam) and then visualized using electrochemiluminescence kit (Beyotime). The results were analyzed using an imaging system (Tanon 5200, Shanghai, China).

### Proliferation detection

Cell vitality was assessed using cell counting kit-8 (CCK-8) (HY-K0301; MedChemExpress, New Jersey, USA). The cells were plated in 96-well plates (1 × 10^4^/well). CCK-8 assay was performed according to the protocol of instructions. The absorbance at 450 nm was then detected under a microplate reader.

### Wound-healing assay

The cells were plated into 6-well plates and scratched using a sterile pipette tip. After washing 3 times with phosphate buffer solution (Thermo Fisher Scientific), cells were added into serum-free medium and incubated in a 5% CO_2_ incubator at 37℃. Images of cells were taken at 0 and 24 h using an inverted microscope and analyzed by ImageJ software to calculate the healing rates of cells in both groups, following the formular: healing ratio (%) = (scratch spacing at 0 h - scratch spacing at 24 h)/ scratch spacing at 0 h × 100%.

### Transwell assay

Cells (cell density 2 × 10^4^/well) were seeded into the top chamber of transwell insert coated with Matrigel (Corning Inc, New York, USA), and 600 µL 20% fetal bovine serum (Thermo Fisher Scientific) medium was added to the bottom chamber for incubation. After 24 h, cells in the lower surface were fixed in 4% paraformaldehyde (Sigma Aldrich, St. Louis, MO, USA) for 30 min and stained with 0.1% crystal violet (Sigma Aldrich) for 20 min. After washing 3 times with phosphate buffer solution, cells were observed and counted using an inverted microscope.

### Statistical analysis

All statistical analysis were conducted using GraphPad Prism 7.0. Data were all represented as mean ± standard deviation. The student’s t-test was used to compare the differences between the two groups. *P* < 0.05 was considered statistically significant.

## Results

### Identification of DEGs

**Fig. 1:**
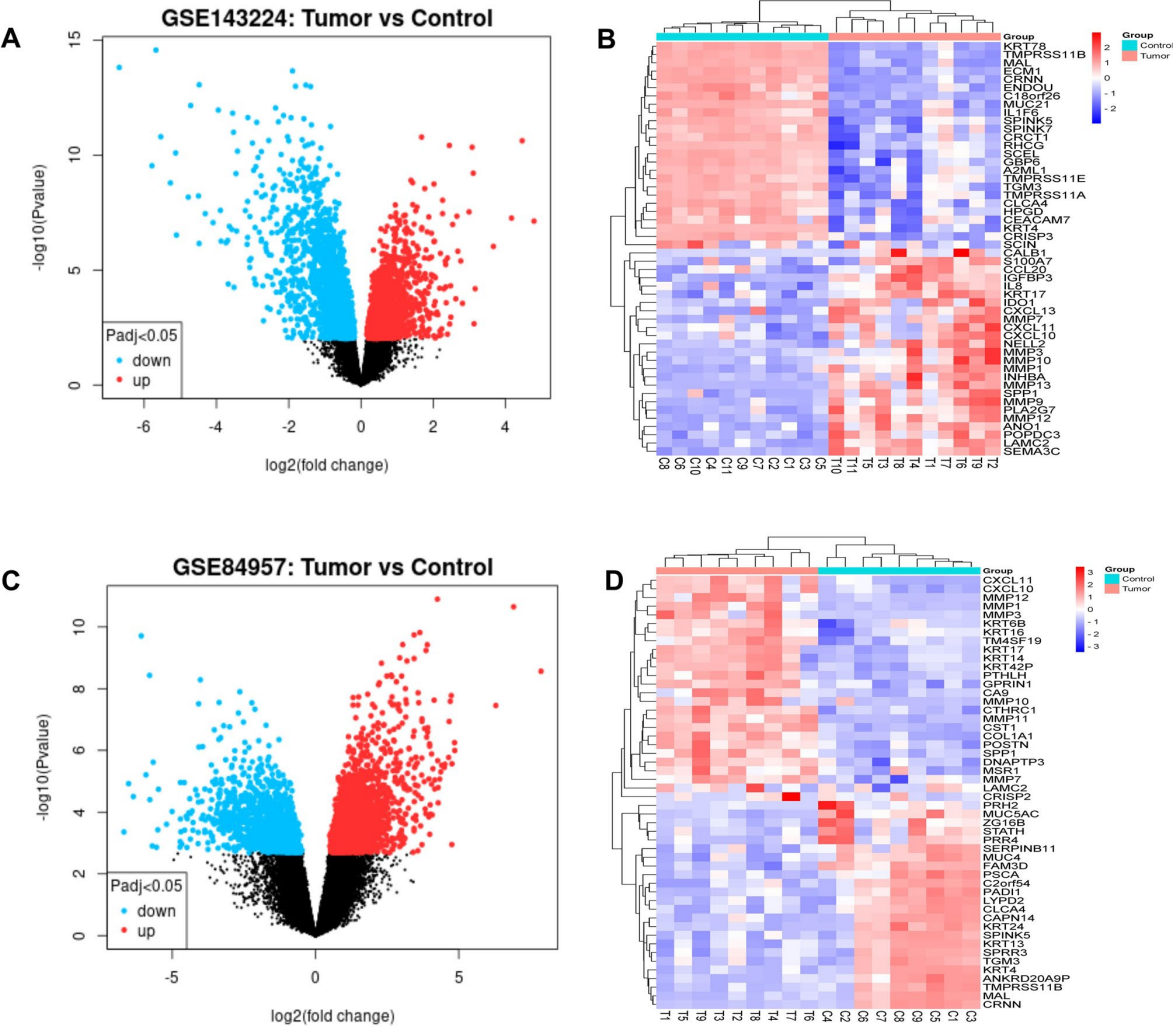
Differentially expressed genes (DEGs) of the GSE143224 and GSE84957 datasets. (**A**) Volcano plot of the DEGs identified from the GSE143224 dataset. (**B**) Heat map of the DEGs identified from the GSE143224 dataset. (**C**) Volcano plots of the DEGs identified from GSE84957 dataset. (**D)** Heat map of the DEGs identified from the GSE84957 dataset

**Fig. 2:**
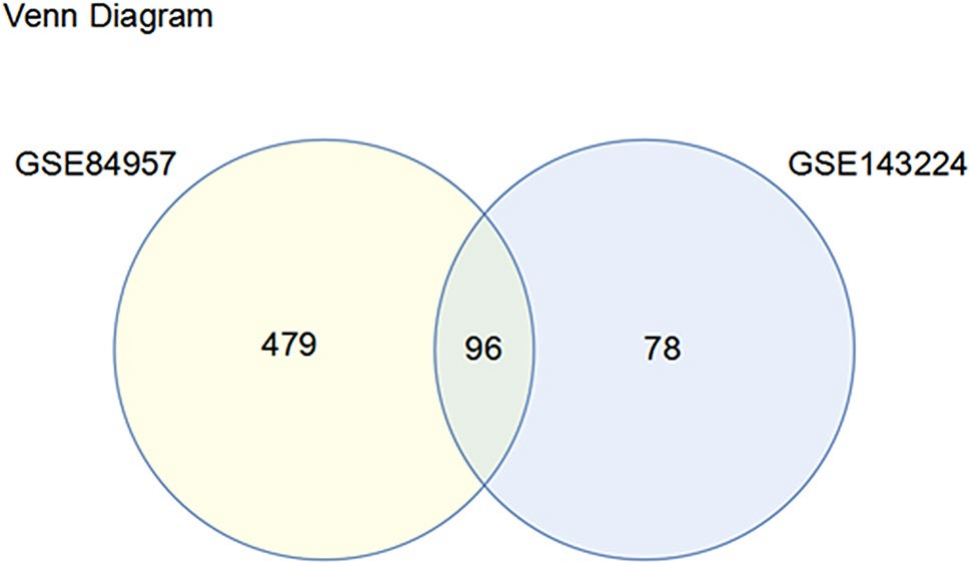
Venn diagrams of common DEGs screened from the GSE143224 and GSE84957 datasets. A total of 96 overlapping DEGs are shown in the crossing section

Total 174 DEGs were screened from GSE143224 dataset, of which 47 genes were upregulated and 127 genes were downregulated. And 575 DEGs were identified from GSE84957 dataset, of which 326 genes were up-regulated and 249 genes were down-regulated. Cluster analysis of the DEGs in the two datasets was carried out to obtain volcano maps, which showed the distribution of DEGs in tumor group compared with the control group (Fig. 1A and C). The heat map suggested that the samples were clustered with high confidence (Fig. 1B and D). Venn diagram revealed that there were 96 common DEGs in the two datasets (Fig. 2). The data correction results revealed that 25 samples in GSE143224 and 18 samples in GSE84957 were obtained (Figure. S1). The top 25 DEGs (upregulated and downregulated) of GSE143224 and GSE84957 datasets were screened out and displayed in Table S2 and S3.

### Functional enrichment analysis

**Fig. 3:**
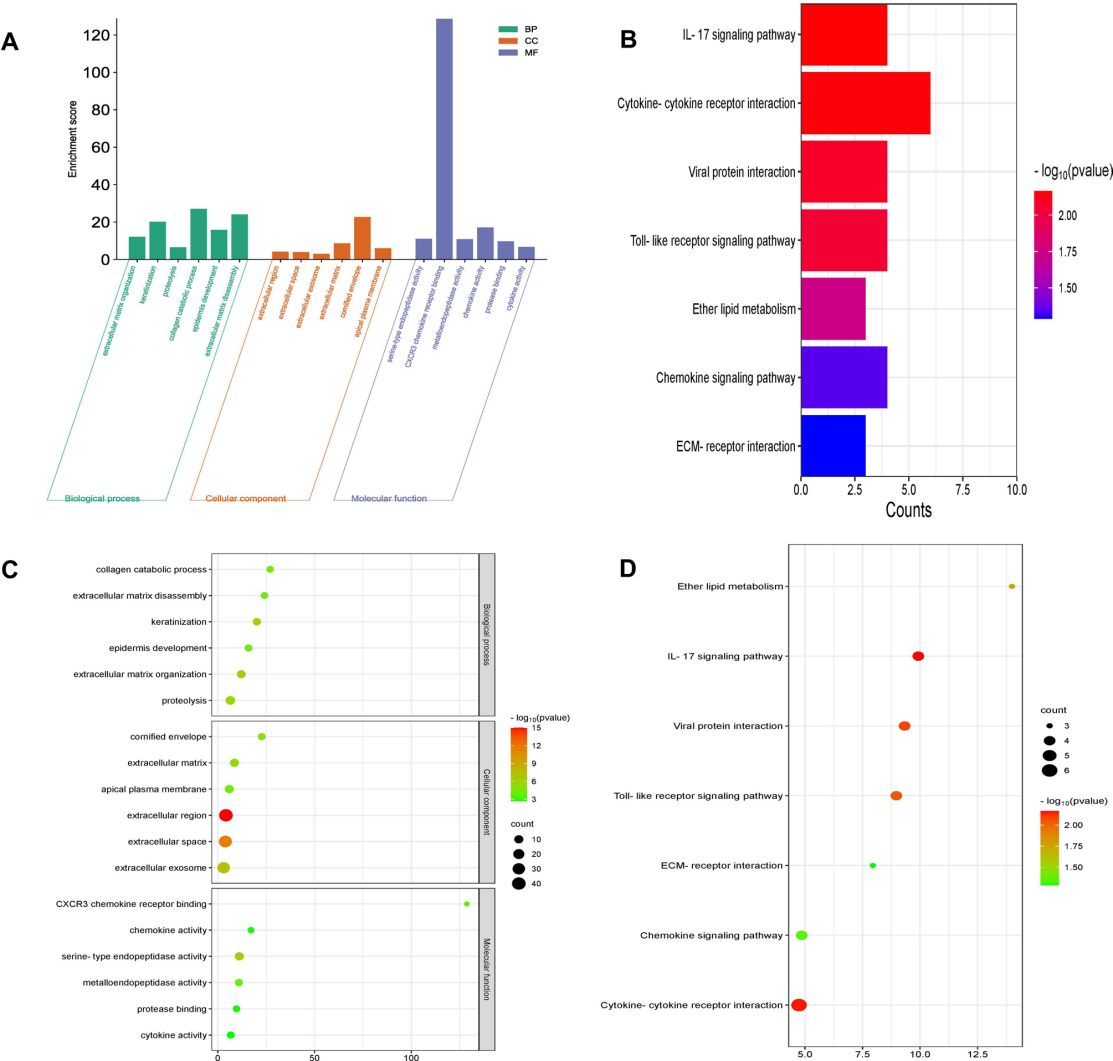
Enrichment analysis of common DEGs. (**A**) Gene Ontology (GO) enrichment bar chart. The abscissa is GO term and the ordinate is -log10 (p-value) of enrichment in each term. (**B**) Kyoto Encyclopedia of Genes and Genomes (KEGG) bar chart; (**C**) GO enrichment analysis bubble map, color depth of each node represents corrected p value, the size of the node refers to the number of genes involved; (**D**) KEGG bubble diagram

The most significantly enriched top 6 GO terms were screened. The DEGs were significantly enriched in “extracellular matrix organization”, “keratinization”, “proteolysis”, “collagen catabolic process”, “epidermis development”, “extracellular matrix disassembly” in BP; “extracellular region”, “extracellular space”, “extracellular exosome”, “extracellular matrix”, “cornified envelope”, “apical pl1asma membrane” in CC; “serine-type endopeptidase activity”, “CXCR3 chemokine receptor binding”, “metalloendopeptidase activity”, “chemokine activity, protease binding”, and “cytokine activity” in MF. The top 6 GO enrichment results were displayed in bar chart and bubble chart (Fig. 3A, C).

KEGG pathway analysis suggested that the enriched pathways mainly included “IL-17 signaling pathway”, “Cytokine-cytokine receptor interaction”, “Viral protein interaction with cytokine and cytokine receptor”, “Toll-like receptor signaling pathway”, “Ether lipid metabolism”, “Chemokine signaling pathway”, “ECM-receptor interaction”. The most significantly enriched KEGG pathways were displayed in bar chart and bubble chart (Fig. 3B, D).

### PPI network construction and identification of hub genes

**Fig. 4:**
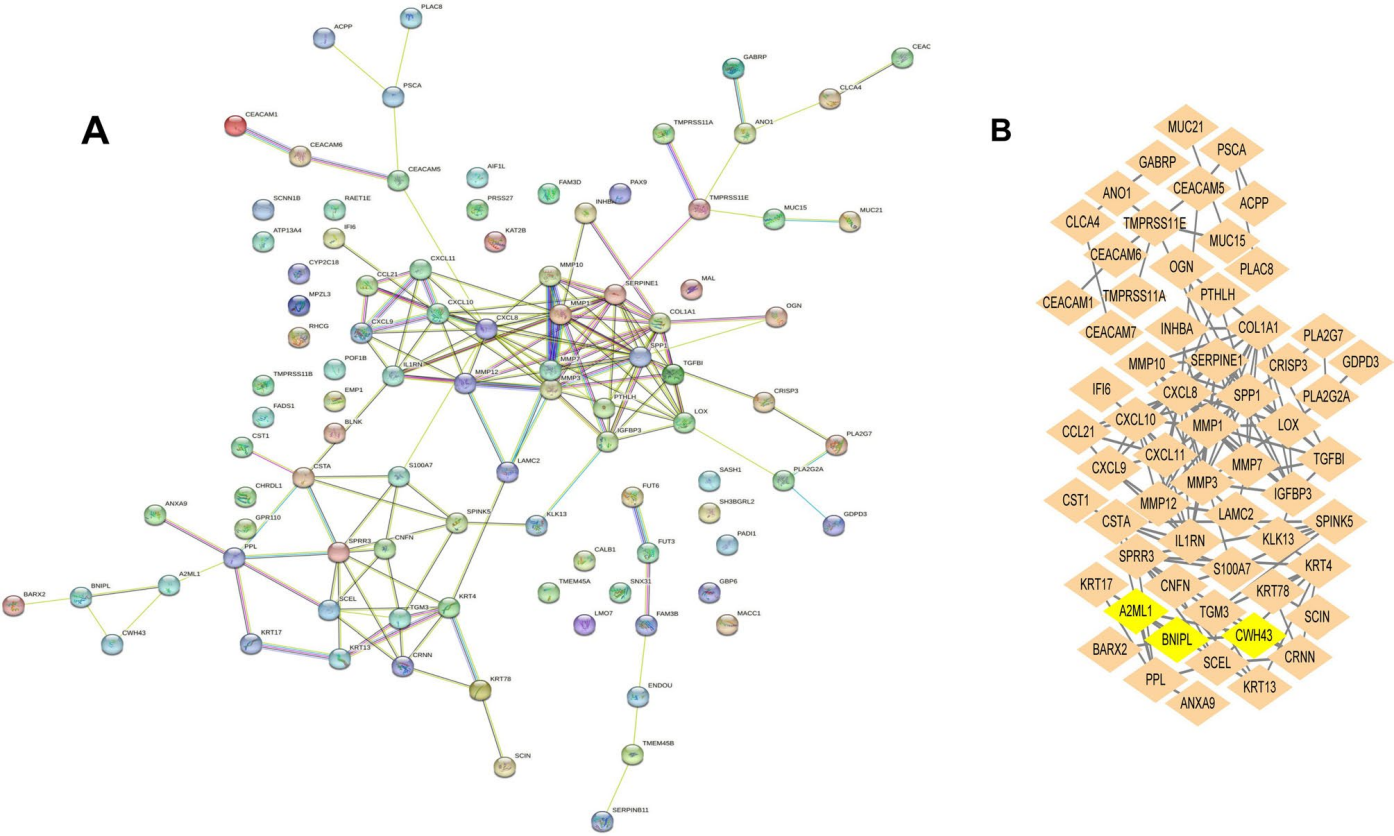
Protein-protein interaction network and the identification of hub genes. (**A**) The protein-protein interaction network of DEGs. (**B**) The key module where BNIPL resides. The lines between the nodes represent the interactions between genes

The PPI networks of DEGs were visualized using Cytoscape (Fig. 4A). We identified 6 hub genes (BNIPL, KRT4, IGFBP3, MMP10, MMP3, and TGFBI) from the key module of PPI networks (Fig. 4B).

**Fig. 5:**
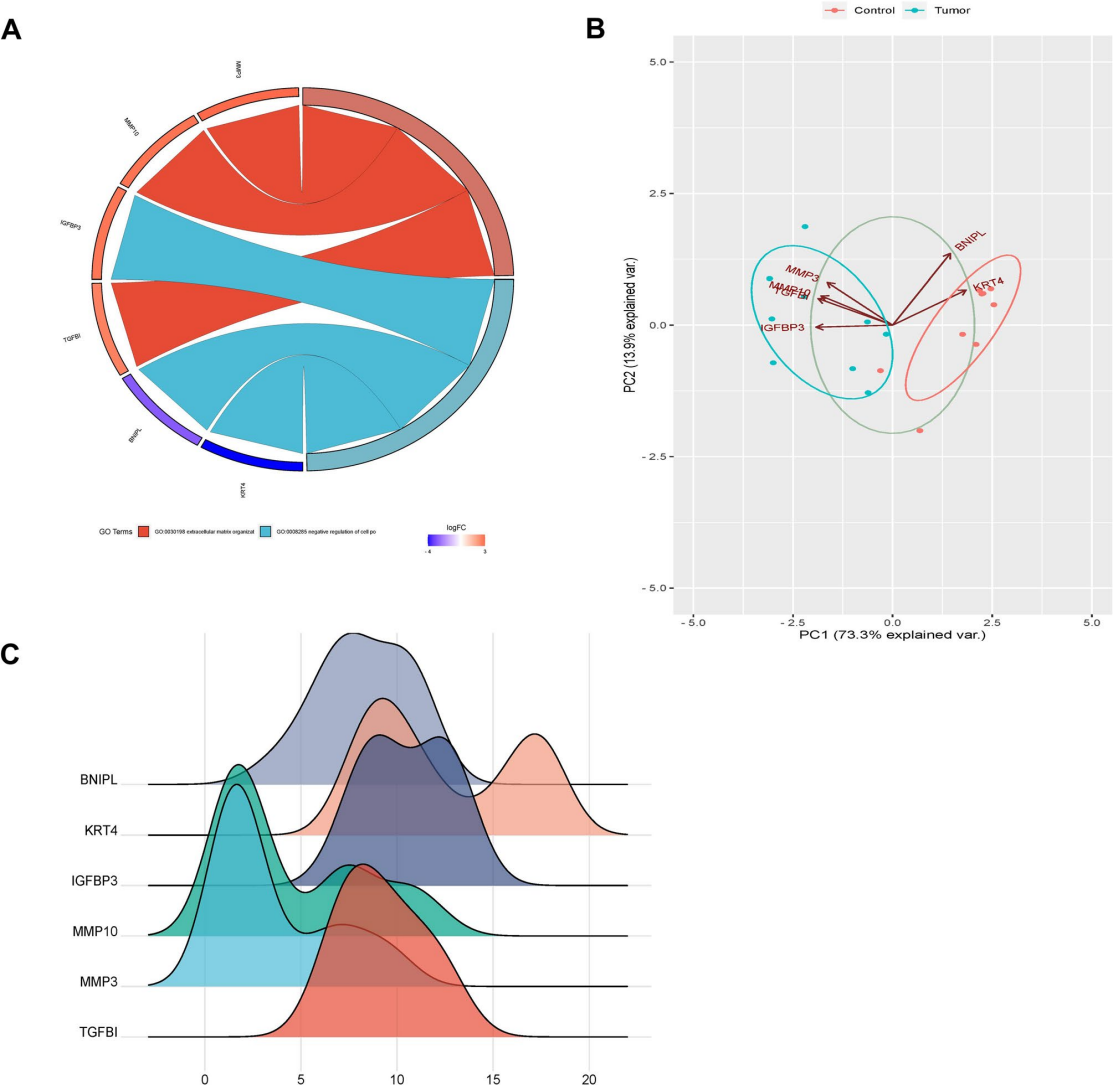
Analysis of hub genes. (**A**) GO enrichment chord map, including three parts: logFC is fold-change of genes; other columns are the GO term, and the different links of the gene indicate whether the gene is in this GO term. (**B**) Principal component analysis of hub genes. PC1 and PC2 on the axes in the figure are the first and second principal components (the variance explanation rate of differences by potential variables); Dots represent samples, and different colors represent different groups. (**C**) Expression of ridgeline plot of hub gene. The horizontal coordinate is the gene expression, the shape of the mountain represents the dispersion between a set of data, and the height is the number of samples corresponding to the gene expression

The GO enrichment results that the hub genes enriched in were mainly “extracellular matrix” and “negative regulation of cell proliferation” (Fig. 5A). PCA analysis showed that the variance interpretation rate of PC1 and PC2 was 87.2%, indicating that hub genes could distinguish between the LC samples from control samples. The scatter plot suggested that the two groups had a good separation, further confirming the validity of the two principal components (Fig. 5B). The expression levels of hub genes in the original sample of GSE84957 dataset were shown in the ridgeline plot (Fig. 5C).

### Analysis of hub genes

**Fig. 6:**
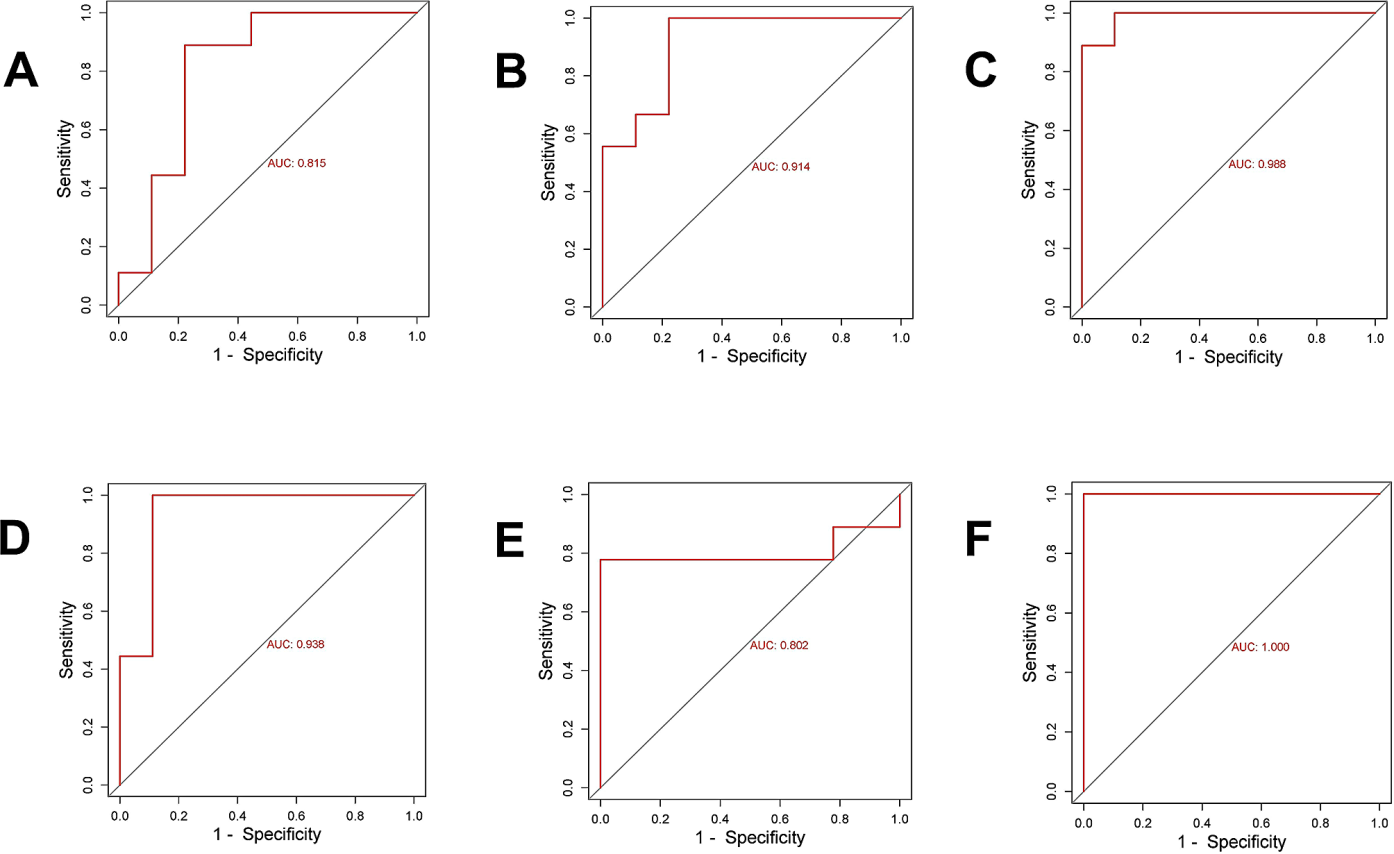
Receiver operating characteristic curves of hub genes. (**A**) ROC curves are plotted using expression of BNIPL in GSE84957dataset. (**B**) ROC curves are plotted using the expression of KRT4 in GSE84957 dataset. (**C**) ROC curves are plotted using the expression of IGFBP3 in GSE84957 dataset. (**D**) ROC curves are plotted using the expression of MMP10 in GSE84957 dataset. (**E**) ROC curves are plotted using the expression of MMP3 in GSE84957 dataset. (**F**) ROC curves are plotted using the expression of TGFB1 in GSE84957 dataset. The horizontal coordinate is false positive rate and the vertical coordinate is true positive rate, indicating the expression level of the gene in the sample data

**Fig. 7:**
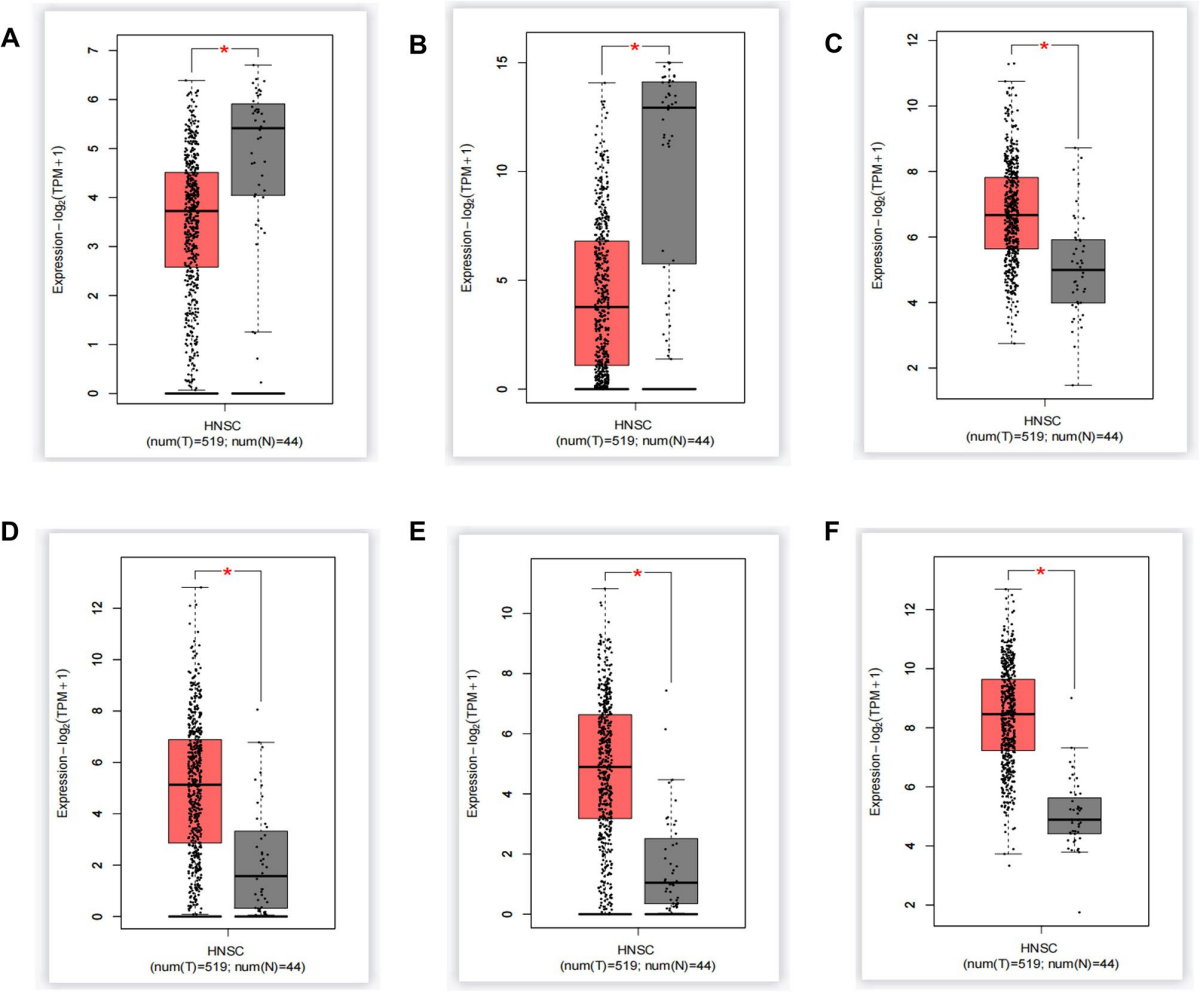
The expression of hub genes (BNIPL, KRT4, IGFBP3, MMP10, MMP3, TGFBI) is verified using Gene Expression Profile Interaction Analysis 2 database. (**A**) BNIPL (**B**) KRT4 (**C**) IGFBP3 (**D**) MMP10 (**E**) MMP3 (**F**) TGFBI. ^*^P < 0.05* vs. *normal tissues

We plotted the ROC curves of BNIPL, KRT4, IGFBP3, MMP10, MMP3, and TGFBI using the raw data of GSE84957 dataset (Fig. 6). The false positive rates of ROC curves of BNIPL, KRT4, IGFBP3, MMP10, MMP3 and TGFB were 18.5%, 8.6%, 1.2%, 6.2%, 19.8%, and 0%, respectively while the true positive rates were 81.5%, 91.4%, 98.9%, 93.8%, 80.2%, and 100%, respectively. Compared with normal group, the expression levels of BNIPL and KRT4 were downregulated, while IGFBP3, MMP10, MMP3, and TGFBI were upregulated in LC tissues, which were same as the analysis results in the GEO datasets (Fig. 7A-F).

### Analysis of BNIPL in LC

**Fig. 8:**
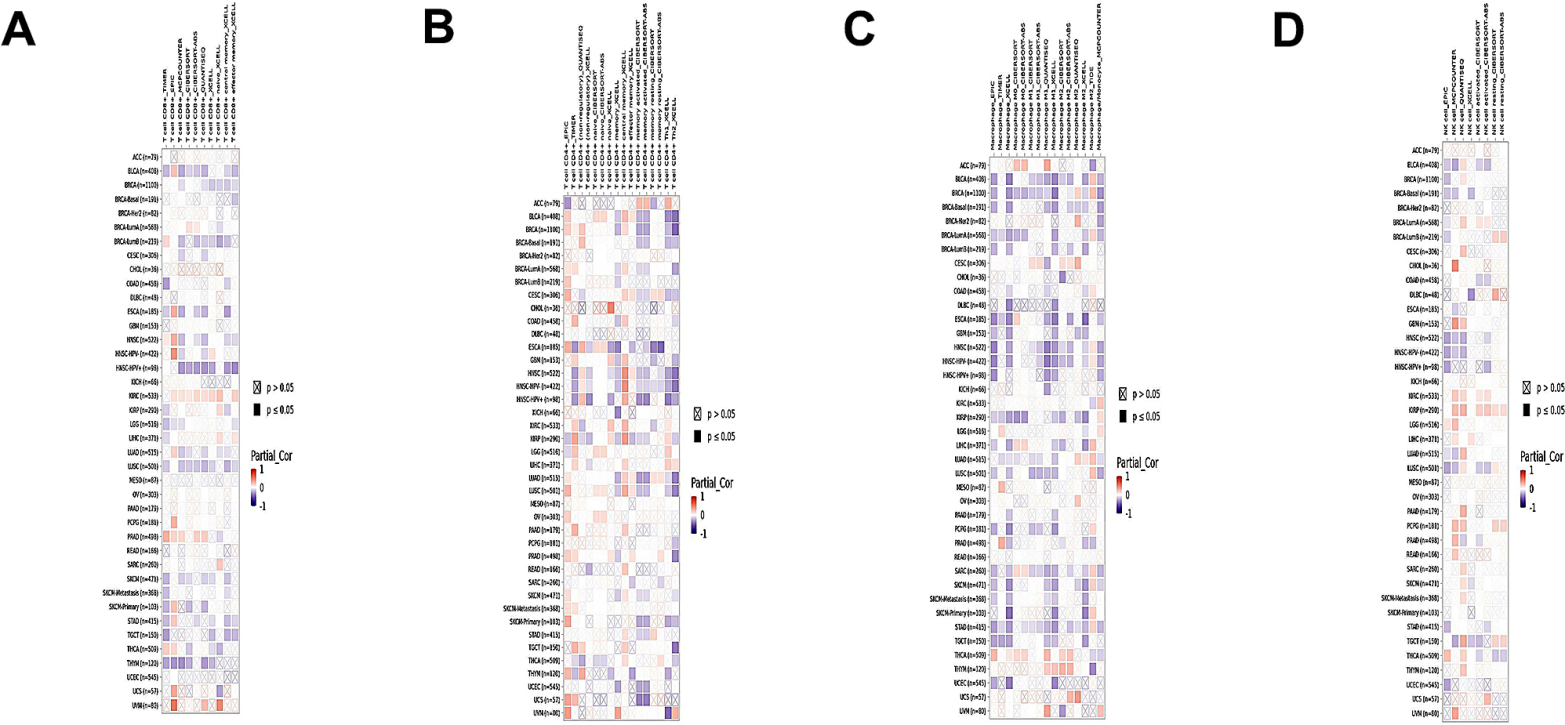
Correlation of BNIPL with immune infiltration. (**A**) Correlation of BNIPL expression with CD8+. (**B**) Correlation of BNIPL expression with CD4+. (**C**) Correlation of BNIPL expression with Macrophage. (**D**) Correlation of BNIPL expression with natural killer cell

Studies have widely reported that KRT4, IGFBP3, MMP10, MMP3, and TGFBI are involved in the pathogenesis of LC and other cancers [15–19]. There are few reports focusing on the role of BNIPL in cancers. In present study, we selected BNIPL as the target gene for further analysis. Kaplan-Meier survival curve reflected significant differences of survival in patients with high and low expression (Figure. S2). BNIPL expression was significantly correlated with the infiltration of the immune cells in LC (Figure. S3). Low expression level of BNIPL had a significant association with CD8+, CD4+, natural killer cell, and Macrophage, which promoted the immune infiltration (Fig. 8A-D).

### Validation of the hub genes expression

**Fig. 9:**
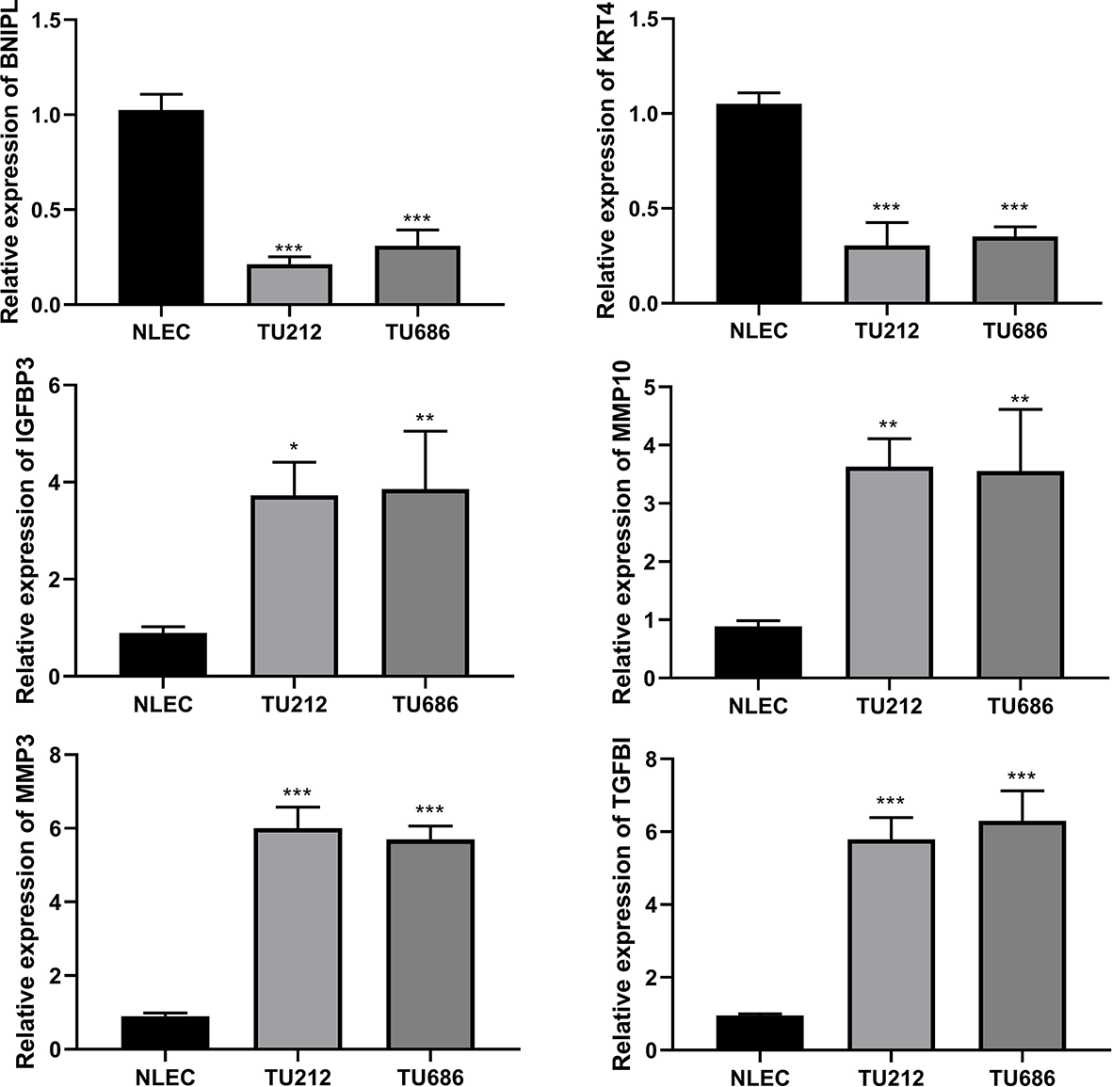
The mRNA expression of BNIPL, KRT4, IGFBP3, MMP10, MMP3, and TGFBI in cells. The mRNA expression levels of BNIPL, KRT4, IGFBP3, MMP10, MMP3, and TGFBI in TU212 and TU686 cells were detected by quantitative real time polymerase chain reaction. ^*^P < 0.05, ^**^P < 0.01, ^***^P < 0.001 *vs*. normal laryngeal epithelial cells (*n* = 3)

qRT-PCR results suggested that the mRNA expression levels of BNIPL and KRT4 in TU212 and TU686 cells were significantly lower than those in NLECs, while the levels of IGFBP3, MMP10, MMP3 and TGFBI were significantly higher than those in NLECs, which was consist with the bioinformatics analysis results (Fig. 9).

### BNIPL inhibits viability, migration, and invasion of LC cells

**Fig. 10:**
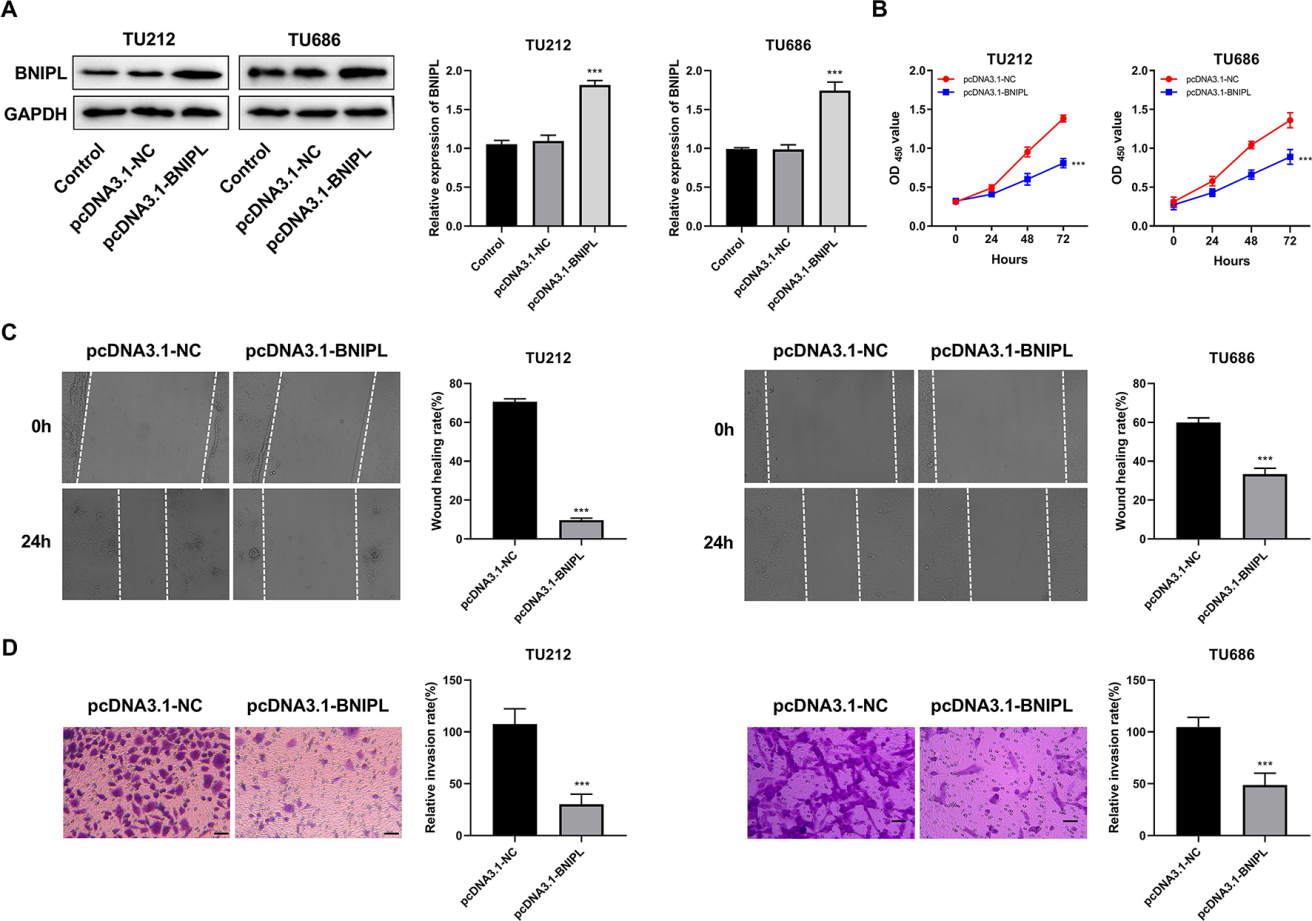
BNIPL inhibits the viability, migration, and invasion of TU212 and TU686 cells. (**A**) The transfection efficiency of BNIPL was detected by western blot. (**B**) The cell viability of TU212 and TU686 cells was evaluated by Cell Counting Kit-8. (**C**) The migration of TU212 and TU686 cells was assessed by wound-healing assay. (**D**) The invasion of TU212 and TU686 cells was measured by transwell assay. (Amplification: 200×, Scale: 100 μm). ^***^P < 0.001 *vs*. pcDNA3.1-NC group, (*n* = 3)

The results of transfection efficiency suggested the expression levels of BNIPL in TU212 and TU686 cell lines of pcDNA3.1-BNIPL group were significantly increased, compared to the pcDNA3.1-NC group (*P* < 0.001) (Fig. 10A). The CCK-8 results suggested that compared with the pcDNA3.1-NC group, the cell viability of TU212 and TU686 cells in pcDNA3.1-BNIPL group was significantly decreased (Fig. 10B). Additionally, the transwell and wound-healing assays revealed that the migration and invasion of TU212 and TU686 cells in the pcDNA3.1-BNIPL group were significantly suppressed, compared with the pcDNA3.1-NC group (Fig. 10C and D).

## Discussion

Poor prognosis induced by invasion and metastasis of tumor cells is the prevalent issues of advanced and metastatic LC [20]. Herein, we identified 6 hub genes (BNIPL, KRT4, IGFBP3, MMP10, MMP3, and TGFBI) of LC with high predictive values using bioinformatics analysis. BNIPL was selected as the key gene, which was correlated with immune cells and promoted the infiltration of immune cell. Moreover, BNIPL was downregulated in LC cells, and overexpression of BINPL inhibited the proliferation, migration, and invasion of LC cells.

Many biomarkers involved in the regulation of malignant tumors have been screened out in previous studies. KRT4 is identified as a hub gene of head and neck squamous cell carcinoma (HNSCC) using CytoHubba after conducting weighted gene co-expression network analysis and differential gene expression analysis between HNSCC and normal tissues [21]. IGFBP3 is a regulated gene of p53 tumor suppressor [22]. IGFBP3 is identified as a biomarker with significant diagnostic value for early esophagogastric junction adenocarcinoma after ROC curve analysis and consistency index evaluation of the prediction value of line chart [23]. After analyzing the data related to oral cancer using the Cancer Genome Atlas, Oncomine, and Kaplan-Meier mapper, the MMP3 and MMP10 are identified as highly altered hub genes of oral cancer [24]. TGFBI is identified as one of the hub genes for potential prognostic relevance of breast cancer by conducting weighted gene co-expression network analysis and univariate Cox regression [25]. In present research, BNIPL, IGFBP3, MMP10, MMP3, and TGFBI was identified as the hub genes of LC from PPI networks. Overall, BNIPL, IGFBP3, MMP10, MMP3, and TGFBI play crucial roles in the development of LC.

KRT4 inhibits the development of oral squamous cell carcinoma and the expression of KRT4 is downregulated due to m6A methylation at the exon-intron boundary preventing intron splicing of KRT4 pre-mRNA in oral squamous cell carcinoma [15]. In patients with LC, serum IGFBP3 levels are significantly higher in the patient group and expresses high diagnostic sensitivity (81.4%) and specificity (80%) [26]. MMP1, MMP3, and MMP10 are highly expressed in HNSCC, compared to other cancers by analyzing the data of Oncomine and GEPIA databases. Our findings suggested BNIPL, IGFBP3, MMP10, MMP3, and TGFBI had high sensitivity and feasibility as hub genes for identifying LC. In conclusion, BNIPL, IGFBP3, MMP10, MMP3, and TGFBI showed good capacities for LC diagnosis.

It has been reported that high levels of T cells infiltration in the tumor microenvironment (TME) have diagnostic value for cancers, which plays a crucial role in tumor cells proliferation, invasion, and migration [27, 28]. Study has reported that infiltration of immune cell in TME is related to clinical prognosis of LC patients: response to induction chemotherapy is better due to less macrophage infiltration and more T cell infiltration in TME [29]. In LC, TNNT3, TNNI2, Desmin, matrix metallopeptidase 9, and cytotoxic T lymphocyte antigen 4 are positively correlated with macrophages and dendritic cells, while negatively correlated with CD4 + T cells [30]. In our research, the low expression of BNIPL had a significant correlation with the expression of T cells (CD8 + and CD4+) and was correlated with other immune cells in TME. In brief, BNIPL can promote infiltration of immune cell in the TME of LC.

Several studies investigate the effect of BNIPL-related molecules on cancer cell function: growth, migration, and invasion by regulating expression levels. In Hep3B cells, BNIPL-1 upregulates the expression of p16INK4, interleukin-12, TRAIL, and lymphotoxin β genes involved in growth inhibition or apoptosis, and downregulates PTEN expression involved in cell proliferation, which suggests that BNIPL-1 may inhibit Hep3B cells growth through cell cycle arrest or apoptotic cell death pathways [31]. In BNIPL-2 transfected Hep3B-Tet-on cells, there are 8 genes involved in cell apoptosis and growth inhibition up-regulating and 7 genes involved in cell proliferation down-regulating, indicating that BNIPL-2 induces apoptosis by regulating the expression of genes involved in apoptosis, growth inhibition, and cell proliferation [32]. Poor prognosis induced by ERBB2 can alter the BNIPL expression, causing an anti-apoptotic phenotype [33]. In addition, overexpression of CD44 restores the cell proliferation suppressed by BNIPL‑2 and BNIPL-2, which can promote migration, invasion, and metastasis of colorectal cancer cells via CD44 [34]. In our research, overexpression of BNIPL inhibited the proliferation, migration, and invasion of LC cells. Study has concluded that BNIPL-2 may be a linker protein located at the front of the Bcl-2 pathway for DNA fragmentation and Cdc42 signaling, and for morphological changes during apoptosis [35]. This inspires us that BNIPL may play an important role in cancer cells in two pathways: regulating DNA breakage and vesicle formation in apoptotic cells. In addition, the interaction of BNIPL with both MIF and GFER proteins maintains homeostasis between cell proliferation and apoptosis [36]. All in all, BNPL exerts an inhibitory effect on the progression of LC.

Taken together, we identified 6 hub genes based on bioinformatics analysis, which had diagnostic and predictive values of LC. In addition, we demonstrated that BNIPL could promote immune cells infiltration in LC and had an inhibitory effect on the proliferation, migration, and invasion of LC cells. Our findings indicate that BNIPL is a promising molecular target and a potential therapeutic for LC, which can provide a new insight for diagnosis and targeted therapy of LC.

### Electronic supplementary material

Below is the link to the electronic supplementary material.

**Supplementary Material 1:** Supplementary Tables (Table S1-S3)

**Supplementary Material 2:** Supplementary Figures (Figure S1-S3)

## Data Availability

The datasets generated and/or analysed during the current study are available in the [National Center for Biotechnology Information database] repository, (https://www.ncbi.nih.gov/geo/)

## References

[1] Zhao Y, Qin J, Qiu Z, Guo J, Chang W. Prognostic role of neutrophil-to-lymphocyte ratio to laryngeal squamous cell carcinoma: a meta-analysis. Braz J Otorhinolaryngol. 2020. 10.1016/j.bjorl.2020.09.015 PMC9483932 33272836

[2] Shah JP, Karnell LH, Hoffman HT, Ariyan S, Brown GS, Fee WE, Glass AG, Goepfert H, Ossoff RH, Fremgen A (1997). Patterns of Care for Cancer of the Larynx in the United States. Arch Otolaryngol Head Neck Surg.

[3] Psychogios G, Karatzanis, Alexander D. Waldfahrer, Frank, Kapsreiter, Markus, Iro. Management of locally advanced laryngeal cancer. Le Journal D’oto Rhino Laryngologie Et De Chirurgie Cervico Faciale; 2014. 10.1186/1916-0216-43-4 PMC3909348 24472173

[4] Vukelić J, Dobrila-Dintinjana R, Marijić B, Maržić D, Braut T, CLINICAL COURSE OF, THE DISEASE AND TREATMENT OUTCOME IN PATIENTS WITH MALIGNANT LARYNGEAL TUMOR (2022). RETROSPECTIVE FIVE-YEAR ANALYSIS. Acta Clin Croat.

[5] Lechien JR, Sadoughi B, Hans S (2022). Laryngeal cancers in paediatric and young adult patients: epidemiology, biology and treatment. Curr Opin Otolaryngol Head Neck Surg.

[6] Siegel RL, Miller KD, Jemal A (2016). Cancer statistics, 2016. CA Cancer J Clin.

[7] Brandstorp-Boesen J, Sørum Falk R, Folkvard Evensen J, Boysen M, Brøndbo K (2016). Risk of recurrence in Laryngeal Cancer. PLoS ONE.

[8] Robinson, Peter N (2014). Genomic data sharing for translational research and diagnostics. Genome Med.

[9] Casotti MC, Meira DD, Alves LNR, Bessa BGO, Campanharo CV, Vicente CR, Aguiar CC, Duque DA, Barbosa DG, Santos E et al. Translational Bioinformatics Applied to the study of Complex diseases. Genes (Basel) 2023; 14. 10.3390/genes14020419 PMC9956936 36833346

[10] Li Y, Cai Q, Shen X, Chen X, Guan Z (2021). Overexpression of B7-H3 is Associated with Poor Prognosis in Laryngeal Cancer. Front Oncol.

[11] Chen J, Luo J, He J, Jiang X, Jiang N, Yang C, Zhong S. Cell Cycle-Related Gene SPC24: A Novel Potential Diagnostic and Prognostic Biomarker for Laryngeal Squamous Cell Cancer. Biomed Res Int. 2023; 2023:1733100. 10.1155/2023/1733100 PMC9884166 36718148

[12] Shen L, Jian H, Hong L, Ming W, Qin W, Wan D, Li YY, Gu J (2003). The apoptosis-associated protein BNIPL interacts with two cell proliferation-related proteins, MIF and GFER. FEBS Lett.

[13] Xie L, Qin W, Li J, He X, Zhang H, Yao G, Shu H, Yao M, Wan D, Gu J (2007). BNIPL-2 promotes the invasion and metastasis of human hepatocellular carcinoma cells. Oncol Rep.

[14] Qin W, Hu J, Guo M, Xu J, Li J, Yao G, Zhou X, Jiang H, Zhang P, Shen L (2003). BNIPL-2, a novel homologue of BNIP-2, interacts with Bcl-2 and Cdc42GAP in apoptosis. Biochem Biophys Res Commun.

[15] Li X, Fang J, Tao X, Xia J, Cheng B, Wang Y (2023). Splice site m6A methylation prevents binding of DGCR8 to suppress KRT4 pre-mRNA splicing in oral squamous cell carcinoma. PeerJ.

[16] Gill ZP, Perks CM, Newcomb PV, Holly J (1997). Insulin-like growth factor-binding protein (IGFBP-3) predisposes breast Cancer cells to programmed cell death in a Non-IGF-dependent manner. J Biol Chem.

[17] Deraz EM, Kudo Y, Yoshida M, Obayashi M, Takata T (2011). MMP-10/Stromelysin-2 promotes Invasion of Head and Neck Cancer. PLoS ONE.

[18] Zhang C, Li C, Zhu M, Zhang Q, Xie Z, Gang N, Song X, Jin L, Li G, Zheng H (2013). Meta-analysis of MMP2, MMP3, and MMP9 promoter polymorphisms and Head and Neck Cancer Risk. PLoS ONE.

[19] Liu W, Xu Y, Bai S, Liao L (2023). Bioinformatics analysis of key biomarkers for bladder cancer. Biomed Rep.

[20] Weinberg RA, Chaffer CL (2011). A perspective on Cancer Cell Metastasis. Science.

[21] Li CY, Cai JH, Tsai JJP, Wang CCN (2020). Identification of hub genes Associated with Development of Head and Neck Squamous Cell Carcinoma by Integrated Bioinformatics Analysis. Front Oncol.

[22] Cai Q, Dozmorov M, Oh Y. IGFBP-3/IGFBP-3 receptor system as an Anti-tumor and Anti-metastatic Signaling in Cancer. Cells 2020; 9. 10.3390/cells9051261 PMC7290346 32443727

[23] Ding TY, Peng YH, Hong CQ, Huang BL, Liu CT, Luo Y, Chu LY, Zhang B, Li XH, Qu QQ (2022). Serum insulin-like growth factor binding protein 3 as a promising diagnostic and prognostic biomarker in esophagogastric junction adenocarcinoma. Discov Oncol.

[24] Thakore VP, Patel KD, Bhadresha KP, Patel PS, Jain NK (2022). An integrative analysis to enumerate candidate genes for clinical use in oral cancer. J Cancer Res Ther.

[25] Fan TD, Bei DK, Li SW. Nomogram Models Based on the Gene Expression in Prediction of Breast Cancer Bone Metastasis. J Healthc Eng. 2022; 2022:8431946. 10.1155/2022/8431946 PMC9424032 36046013

[26] Pamuk AE, Gedik ME, Sutay Suslu N, Gunaydin G. Candidate angiogenesis-related biomarkers in patients with laryngeal carcinoma (AngLaC): a prospective cohort study. Otolaryngol Head Neck Surg; 2023. 10.1002/ohn.219 36939422

[27] Lei Y, Xie Y, Tan YS, Prince ME, Moyer JS, N?R J, Wolf GT. Telltale tumor infiltrating lymphocytes (TIL) in oral, head & neck cancer. Oral Oncol 2016:159–65. 10.1016/j.oraloncology.2016.08.003 PMC5257271 27553942

[28] Martino JD, Mondal C, Bravo-Cordero JJ (2019). Textures of the tumour microenvironment. Essays Biochem.

[29] Karpathiou G, Casteillo F, Giroult JB, Forest F, Peoc’H M. Prognostic impact of immune microenvironment in laryngeal and pharyngeal squamous cell carcinoma: Immune cell subtypes, immuno-suppressive pathways and clinicopathologic characteristics. Oncotarget 2017; 8-. 10.18632/oncotarget.14242 PMC5386686 28038471

[30] Han X, Cheng X, Dai K, Bao W, Ding R, Wan Y (2023). Identification of immunocell infiltrates and effective diagnostic biomarkers in laryngeal carcinoma. Med (Baltim).

[31] Xie L, Qin WX, Li JJ, He XH, Shu HQ, Yao GF, Wan DF, Gu JR (2005). cDNA expression array analysis of gene expression in human hepatocarcinoma Hep3B cells induced by BNIPL-1. Acta Biochim Biophys Sin (Shanghai).

[32] Xie L, Qin WX, He XH, Shu HQ, Yao GF, Wan DF, Gu JR (2004). Differential gene expression in human hepatocellular carcinoma Hep3B cells induced by apoptosis-related gene BNIPL-2. World J Gastroenterol.

[33] Petry IB, Fieber E, Schmidt M, Gehrmann M, Gebhard S, Hermes M, Schormann W, Selinski S, Freis E, Schwender H (2010). ERBB2 induces an antiapoptotic expression pattern of Bcl-2 family members in node-negative breast cancer. Clin Cancer Res.

[34] Gao L, Liu H, Yin N, Zuo S, Jin G, Hu Y, Hu D, Li Y, Song Q, Fei X (2019). BNIPL–2 expression is correlated with the prognosis and regulates the proliferation of colorectal cancer through CD44. Mol Med Rep.

[35] Qin W, Hu J, Guo M, Xu J, Li J, Yao G, Zhou X, Jiang H, Zhang P, Shen L (2003). BNIPL-2, a novel homologue of BNIP-2, interacts with Bcl-2 and Cdc42GAP in apoptosis. Biochem Biophys Res Commun.

[36] Shen L, Hu J, Lu H, Wu M, Qin W, Wan D, Li YY, Gu J (2003). The apoptosis-associated protein BNIPL interacts with two cell proliferation-related proteins, MIF and GFER. FEBS Lett.

