# Supplementary material for: BNIPL is a promising biomarker of laryngeal cancer: novel insights from bioinformatics analysis and experimental validation
Source: BMC Med Genomics. 2024 Feb 1;17:45. doi: 10.1186/s12920-024-01811-z (PMC10832104; doi:10.1186/s12920-024-01811-z)
Supplement: Supplementary file 1 — Supplementary Material 1: Supplementary Tables (Table S1-S3) [file 12920_2024_1811_MOESM1_ESM.docx]

Table S1 Primer sequence information

| Name | Sequences (5’-3’) |
| --- | --- |
| GAPDH-F | GAGTCAACGGATTTGGTCGT |
| GAPDH-R | TTGATTTTGGAGGGATCTCG |
| BNIPL-F | TGTGGGATGTGACTGGAGAA |
| BNIPL-R | AGGTGTAGTTGGGGATGCTG |
| KRT4-F | CTCCAGCAAAAACCTTGAGC |
| KRT4-R | AAGTCATTCTCGGCTGCTGT |
| IGFBP3-F | CCTGCCGTAGAGAAATGGAA |
| IGFBP3-R | AGGCTGCCCATACTTATCCA |
| MMP10-F | GGCTCTTTCACTCAGCCAAC |
| MMP10-R | TCCCGAAGGAACAGATTTTG |
| MMP3-F | GCAGTTTGCTCAGCCTATCC |
| MMP3-R | GAGTGTCGGAGTCCAGCTTC |
| TGFBI-F | GTGTGTGCTGTGCAGAAGGT |
| TGFBI-R | TTGAGAGTGGTAGGGCTGCT |

Table. S2 Top 25 DEGs of GSE143224

| Name | Description | log2FoldChange | pval | up/down |
| --- | --- | --- | --- | --- |
| MMP1 | matrix metallopeptidase 1 (interstitial collagenase) | 4.79 | 7.41E-08 | up |
| MMP12 | matrix metallopeptidase 12 (macrophage elastase) | 4.46 | 2.37E-11 | up |
| MMP13 | matrix metallopeptidase 13 (collagenase 3) | 4.16 | 5.47E-08 | up |
| SPP1 | secreted phosphoprotein 1 | 3.66 | 9.38E-07 | up |
| MMP7 | matrix metallopeptidase 7 (matrilysin, uterine) | 3.16 | 6.50E-05 | up |
| S100A7 | S100 calcium binding protein A7 | 3.13 | 2.17E-03 | up |
| LAMC2 | laminin, gamma 2 | 3.11 | 6.07E-10 | up |
| SEMA3C | sema domain, immunoglobulin domain (Ig), short basic domain, secreted, (semaphorin) 3C | 3.08 | 4.52E-11 | up |
| ANO1 | anoctamin 1, calcium activated chloride channel | 2.99 | 2.94E-08 | up |
| CXCL13 | chemokine (C-X-C motif) ligand 13 | 2.81 | 2.80E-04 | up |
| IL8 | interleukin 8 | 2.76 | 4.01E-06 | up |
| INHBA | inhibin, beta A | 2.68 | 1.51E-06 | up |
| POPDC3 | popeye domain containing 3 | 2.65 | 4.56E-08 | up |
| MMP3 | matrix metallopeptidase 3 (stromelysin 1, progelatinase) | 2.62 | 1.78E-04 | up |
| PLA2G7 | phospholipase A2, group VII (platelet-activating factor acetylhydrolase, plasma) | 2.54 | 1.04E-07 | up |
| CXCL10 | chemokine (C-X-C motif) ligand 10 | 2.49 | 1.11E-03 | up |
| MMP10 | matrix metallopeptidase 10 (stromelysin 2) | 2.48 | 4.85E-05 | up |
| CXCL11 | chemokine (C-X-C motif) ligand 11 | 2.45 | 2.23E-03 | up |
| IGFBP3 | insulin-like growth factor binding protein 3 | 2.44 | 3.80E-11 | up |
| KRT17 | keratin 17 | 2.39 | 2.96E-06 | up |
| CALB1 | calbindin 1, 28kDa | 2.39 | 6.16E-03 | up |
| CCL20 | chemokine (C-C motif) ligand 20 | 2.36 | 2.63E-04 | up |
| NELL2 | NEL-like 2 (chicken) | 2.35 | 5.79E-06 | up |
| IDO1 | indoleamine 2,3-dioxygenase 1 | 2.35 | 5.42E-05 | up |
| MMP9 | matrix metallopeptidase 9 | 2.33 | 4.10E-06 | up |
| TMPRSS11B | transmembrane protease, serine 11B | -6.69 | 1.53E-14 | down |
| MUC21 | mucin 21, cell surface associated | -5.78 | 2.88E-10 | down |
| CRNN | cornulin | -5.67 | 2.68E-15 | down |
| CRISP3 | cysteine-rich secretory protein 3 | -5.54 | 1.58E-11 | down |
| TGM3 | transglutaminase 3 (E polypeptide, protein-glutamine-gamma-glutamyltransferase) | -5.27 | 1.61E-09 | down |
| MAL | mal, T-cell differentiation protein | -5.13 | 8.02E-11 | down |
| SPINK7 | serine peptidase inhibitor, Kazal type 7 (putative) | -5.11 | 2.99E-07 | down |
| CLCA4 | chloride channel accessory 4 | -4.78 | 6.74E-09 | down |
| C18orf26 | chromosome 18 open reading frame 26 | -4.71 | 6.93E-13 | down |
| SCEL | sciellin | -4.50 | 5.96E-09 | down |
| KRT78 | keratin 78 | -4.48 | 8.70E-14 | down |
| TMPRSS11E | transmembrane protease, serine 11E | -4.48 | 7.00E-07 | down |
| CRCT1 | cysteine-rich C-terminal 1 | -4.31 | 3.52E-08 | down |
| SPINK5 | serine peptidase inhibitor, Kazal type 5 | -4.09 | 8.63E-08 | down |
| SCIN | scinderin | -3.95 | 1.10E-12 | down |
| IL1F6 | interleukin 1 family, member 6 (epsilon) | -3.89 | 2.54E-08 | down |
| A2ML1 | alpha-2-macroglobulin-like 1 | -3.87 | 5.52E-07 | down |
| RHCG | Rh family, C glycoprotein | -3.73 | 6.49E-07 | down |
| GBP6 | guanylate binding protein family, member 6 | -3.69 | 5.13E-07 | down |
| TMPRSS11A | transmembrane protease, serine 11A | -3.67 | 4.03E-05 | down |
| HPGD | hydroxyprostaglandin dehydrogenase 15-(NAD) | -3.64 | 1.26E-07 | down |
| KRT4 | keratin 4 | -3.57 | 1.70E-07 | down |
| ECM1 | extracellular matrix protein 1 | -3.55 | 1.50E-12 | down |
| ENDOU | endonuclease, polyU-specific | -3.53 | 1.01E-11 | down |
| CEACAM7 | carcinoembryonic antigen-related cell adhesion molecule 7 | -3.51 | 5.63E-05 | down |

Table. S3 Top 25 DEGs of GSE84957

| Name | Description | log2FoldChange | pval | up/down |
| --- | --- | --- | --- | --- |
| MMP1 | Matrix Metallopeptidase 1 | 4.86 | 1.01E-06 | up |
| SPP1 | Secreted Phosphoprotein 1 | 4.85 | 5.61E-07 | up |
| CXCL11 | C-X-C Motif Chemokine Ligand 11 | 4.76 | 2.70E-06 | up |
| MMP10 | Matrix Metallopeptidase 10 | 4.76 | 1.13E-03 | up |
| CTHRC1 | Collagen Triple Helix Repeat Containing 1 | 4.74 | 1.68E-08 | up |
| COL1A1 | Collagen Type I Alpha 1 Chain | 4.71 | 2.58E-08 | up |
| CXCL10 | C-X-C Motif Chemokine Ligand 10 | 4.52 | 2.82E-06 | up |
| LAMC2 | Laminin Subunit Gamma 2 | 4.50 | 3.55E-06 | up |
| KRT17 | Keratin 17 | 4.40 | 3.04E-06 | up |
| KRT42P | Keratin 42, Pseudogene | 4.28 | 1.60E-05 | up |
| POSTN | Periostin | 4.27 | 2.16E-06 | up |
| CST1 | Cystatin SN | 4.26 | 1.26E-11 | up |
| DNAPTP3 | No results | 4.16 | 9.06E-06 | up |
| MMP12 | Matrix Metallopeptidase 12 | 4.16 | 1.77E-05 | up |
| PTHLH | Parathyroid Hormone Like Hormone | 4.10 | 3.93E-06 | up |
| KRT6B | Keratin 6B | 4.08 | 1.23E-04 | up |
| TM4SF19 | Transmembrane 4 L Six Family Member 19 | 4.05 | 1.31E-06 | up |
| MMP7 | Matrix Metallopeptidase 7 | 4.01 | 1.16E-04 | up |
| CA9 | Carbonic Anhydrase 9 | 4.00 | 5.79E-05 | up |
| MMP3 | Matrix Metallopeptidase 3 | 3.99 | 5.22E-04 | up |
| KRT16 | Keratin 16 | 3.96 | 1.41E-04 | up |
| MSR1 | Macrophage Scavenger Receptor 1 | 3.92 | 2.53E-07 | up |
| MMP11 | Matrix Metallopeptidase 11 | 3.90 | 3.77E-10 | up |
| GPRIN1 | G Protein Regulated Inducer of Neurite Outgrowth 1 | 3.86 | 5.79E-10 | up |
| KRT14 | Keratin 14 | 3.83 | 7.93E-05 | up |
| PRR4 | Proline Rich 4 | -6.69 | 4.40E-04 | down |
| CRNN | Cornulin | -6.52 | 1.19E-05 | down |
| MAL | Mal, T Cell Differentiation Protein | -6.36 | 3.13E-05 | down |
| CRISP2 | Cysteine Rich Secretory Protein 2 | -6.08 | 1.95E-10 | down |
| CLCA4 | Chloride Channel Accessory 4 | -5.92 | 6.19E-06 | down |
| FAM3D | FAM3 Metabolism Regulating Signaling Molecule D | -5.79 | 3.71E-09 | down |
| KRT4 | Keratin 4 | -5.78 | 3.96E-05 | down |
| CAPN14 | Calpain 14 | -5.51 | 3.74E-04 | down |
| TMPRSS11B | Transmembrane Serine Protease 11B | -5.48 | 1.82E-05 | down |
| KRT13 | Keratin 13 | -5.20 | 2.48E-04 | down |
| SPRR3 | Small Proline Rich Protein 3 | -4.95 | 3.51E-03 | down |
| TGM3 | Transglutaminase 3 | -4.80 | 2.20E-03 | down |
| PRH2 | Proline Rich Protein HaeIII Subfamily 2 | -4.73 | 1.37E-03 | down |
| SERPINB11 | Serpin Family B Member 11 | -4.72 | 1.67E-04 | down |
| MUC4 | Mucin 4, Cell Surface Associated | -4.71 | 1.10E-05 | down |
| SPINK5 | Serine Peptidase Inhibitor Kazal Type 5 | -4.70 | 4.32E-04 | down |
| MUC5AC | Mucin 5AC, Oligomeric Mucus/Gel-Forming | -4.58 | 1.11E-05 | down |
| PADI1 | Peptidyl Arginine Deiminase 1 | -4.48 | 1.64E-04 | down |
| PSCA | Prostate Stem Cell Antigen | -4.42 | 1.06E-04 | down |
| ANKRD20A9P | Ankyrin Repeat Domain 20 Family Member A9, Pseudogene | -4.39 | 3.67E-05 | down |
| STATH | Statherin | -4.36 | 2.84E-03 | down |
| KRT24 | Keratin 24 | -4.31 | 9.14E-05 | down |
| C2orf54 | No results | -4.28 | 2.17E-04 | down |
| ZG16B | Zymogen Granule Protein 16B | -4.10 | 1.05E-03 | down |
| LYPD2 | LY6/PLAUR Domain Containing 2 | -4.09 | 4.53E-04 | down |
