# Supplementary material for: BNIPL is a promising biomarker of laryngeal cancer: novel insights from bioinformatics analysis and experimental validation
Source: BMC Med Genomics. 2024 Feb 1;17:45. doi: 10.1186/s12920-024-01811-z (PMC10832104; doi:10.1186/s12920-024-01811-z)

Figure. S1 Correction results of GSE143224 and GSE84957 datasets. Total 25 samples in GSE143224 and 18 samples in GSE84957 are obtained after sample correction.


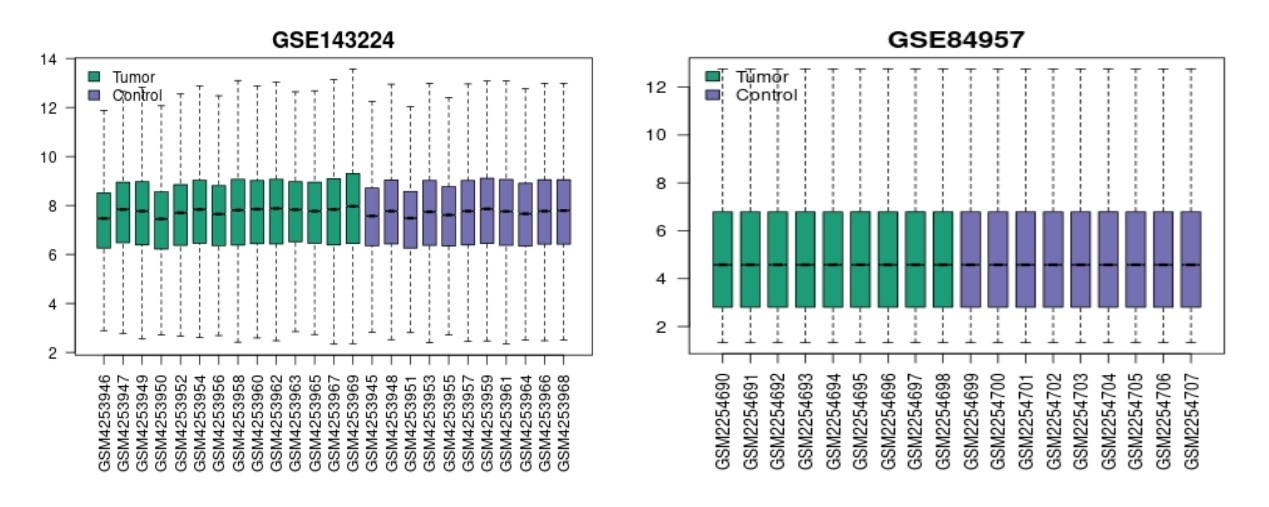


Figure. S2 Kaplan-Meier survival curve. The association between the expression levels of BNIPL and the survival of patients with laryngeal cancer is determined using Kaplan-Meier plotter.


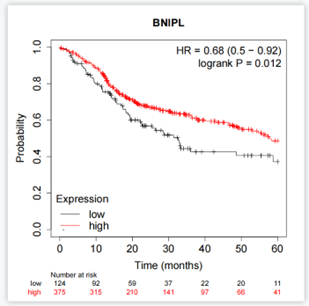


Figure. S3 Correlation between BNIPL and infiltration of immune cell. BNIPL is significantly correlated with the infiltration of the immune cells in laryngeal cancer.


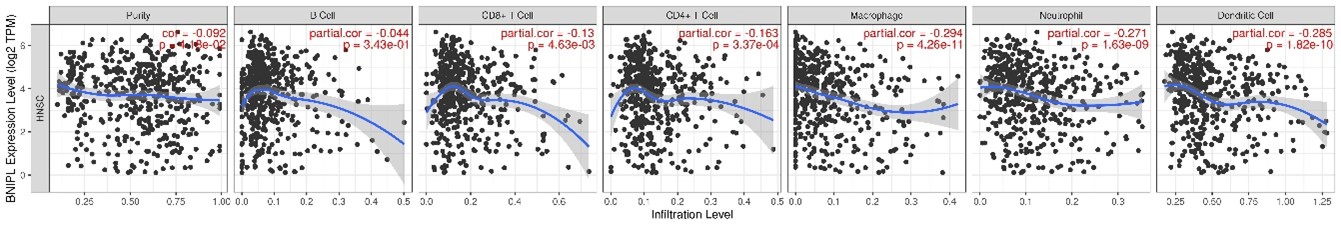

Supplement: Supplementary file 2 — Supplementary Material 2: Supplementary Figures (Figure S1-S3) [file 12920_2024_1811_MOESM2_ESM.docx]
